# Promoter-Wide Hypermethylation of the Ribosomal RNA Gene Promoter in the Suicide Brain

**DOI:** 10.1371/journal.pone.0002085

**Published:** 2008-05-07

**Authors:** Patrick O. McGowan, Aya Sasaki, Tony C. T. Huang, Alexander Unterberger, Matthew Suderman, Carl Ernst, Michael J. Meaney, Gustavo Turecki, Moshe Szyf

**Affiliations:** 1 Department of Psychiatry, Douglas Mental Health University Institute, Montreal, Quebec, Canada; 2 Department of Neurology and Neurosurgery, McGill Program for the Study of Behaviour, Genes and Environment, McGill University, Montreal, Quebec, Canada; 3 Sackler Program for Epigenetics & Psychobiology, McGill University, Montreal, Quebec, Canada; 4 Department of Pharmacology and Therapeutics, McGill University, Montreal, Quebec, Canada; 5 McGill Centre for Bioinformatics, McGill University, Montreal, Quebec, Canada; 6 McGill Group for Suicide Studies, Douglas Mental Health University Institute, Montreal, Quebec, Canada; Deutsches Krebsforschungszentrum, Germany

## Abstract

**Background:**

** Alterations in gene expression in the suicide brain have been reported and for several genes DNA methylation as an epigenetic regulator is thought to play a role. rRNA genes, that encode ribosomal RNA, are the backbone of the protein synthesis machinery and levels of rRNA gene promoter methylation determine rRNA transcription.

**Methodology/Principal Findings:**

We test here by sodium bisulfite mapping of the rRNA promoter and quantitative real-time PCR of rRNA expression the hypothesis that epigenetic differences in critical loci in the brain are involved in the pathophysiology of suicide. Suicide subjects in this study were selected for a history of early childhood neglect/abuse, which is associated with decreased hippocampal volume and cognitive impairments. rRNA was significantly hypermethylated throughout the promoter and 5′ regulatory region in the brain of suicide subjects, consistent with reduced rRNA expression in the hippocampus. This difference in rRNA methylation was not evident in the cerebellum and occurred in the absence of genome-wide changes in methylation, as assessed by nearest neighbor.

**Conclusions/Significance:**

This is the first study to show aberrant regulation of the protein synthesis machinery in the suicide brain. The data implicate the epigenetic modulation of rRNA in the pathophysiology of suicide.

## Introduction

Suicide is a leading cause of death, particularly in males [1], [2]. Although many suicide subjects have a diagnosable psychiatric illness, most persons with a psychiatric disorder never attempt suicide [2]. Suicidal behavior aggregates in families [1], and studies of twins show that monozygotic individuals have a greater concordance for suicide completion and suicide attempts compared to dizygotic individuals [2]–[4]. Non-genetic familial factors, including a history of abuse or neglect during childhood, are also risk factors for suicidal behavior [5], [6]. Similarly, childhood abuse is associated with an increased risk for psychopathology [7], [8] and altered neural development [9].

Several lines of evidence suggest that changes in gene expression in the brain occur in the context of psychiatric disorders and suicide [10]–[15]. Alterations in gene regulation can be caused by epigenetic programming of gene expression in response to environmental exposure, including social and physical adversity [16]. The genome is epigenetically programmed by changes in the chromatin state and by a pattern of modification of the DNA molecule itself through methylation [17]. DNA methylation is a stable epigenetic mark associated with long-lasting silencing of gene expression [18]. In rodents, genes responsive to differences in the quality of maternal care early in life are altered through epigenetic mechanisms [19], [20]. In the human brain, aberrant DNA methylation of specific genes also occurs in the context of psychiatric disorders [21]–[26]. Decreased expression of ribosomal RNA (rRNA), a bottleneck gene for protein production in the cell, occurs in patients with mild cognitive impairment and early Alzheimer's disease [27], [28].

DNA methylation can regulate gene expression in two ways. One is site-specific methylation, involving direct interference with the binding of transcription factors [29]. The second is site-independent promoter-wide methylation, attracting methylated DNA binding proteins and leading to an inactive chromatin structure. In the latter case, the density of methylated CpGs determines the extent of gene silencing [30]. Both mechanisms can regulate rRNA expression. Previous work in cultured mouse cells indicated that rRNA is regulated by methylation of a single CpG dinucleotide at position −133 residing at the upstream control element (UCE) [31]. In human cell culture, the transcriptionally active fraction of rRNA promoters associated with RNA polymerase I (pol I) is completely unmethylated whereas the fraction not associated with pol I is almost completely methylated [32], thus determining transcription by defining the fraction of unmethylated rRNA. Mouse and human rRNA promoters show different CpG densities in the core promoter and UCE (3 in the mouse and 26–28 in the human [31], [33]). Thus, although in both species complete methylation of CpGs in the promoters characterizes inactive alleles, the number of CpGs involved is different suggesting a different mode of regulation by DNA methylation.

In the present study, we tested the hypothesis that rRNA in the human hippocampus of suicide subjects with a history of childhood abuse or severe neglect and controls who died suddenly of unrelated causes without a history of childhood abuse or severe neglect is differentially methylated and expressed. Within the genome there are over 400 copies of the rRNA gene, encoding a large pre-rRNA transcriptional unit (45S) whose expression is tightly regulated by methylation [31]–[34]. We particularly examined the core promoter region and UCE of rRNA because it is involved in the regulation of all pol I transcribed copies of rRNA by methylation [33]. Our strategy was to sample the average methylation pattern of the rRNA promoter at single nucleotide resolution to determine CpG site specificity in the regulation of rRNA gene expression in the brains of suicide subjects and controls. The results implicate the epigenetic modulation of rRNA in the pathophysiology of suicide.

## Results

The subject characteristics are presented in **Table 1**. There were no significant differences in post-mortem interval (PMI), brain pH, or age between suicide subjects and controls (P's>0.05).

**Table 1:** Demographic characteristics and psychiatric diagnoses.^1^

	Suicide		Control	
Male/Female	18/0		12/0	
Age (years)	34 ± 9		36 ± 12	
PMI (hours)	24 ± 5.3		23 ± 5.9	
pH	6.4 ± 0.4		6.5 ± 0.2	
Childhood Abuse/Neglect	18/18	100%	0/12	0%
Mood Disorder	14/18	78%	3/12	25%
Alcohol/Drug Abuse Disorder	12/18	67%	5/12	42%
Anxiety Disorder	3/18	17%	1/12	8%

The values are mean ± SD.

1 The number of subjects in each group represents the total number of subjects used for methylation and expression analysis. Additional subjects used for expression analysis did not differ from the other subjects in any of the listed measures (see Materials and Methods for details; P's>0.05).

### Genotyping of the rRNA promoter

**Figure 1:**
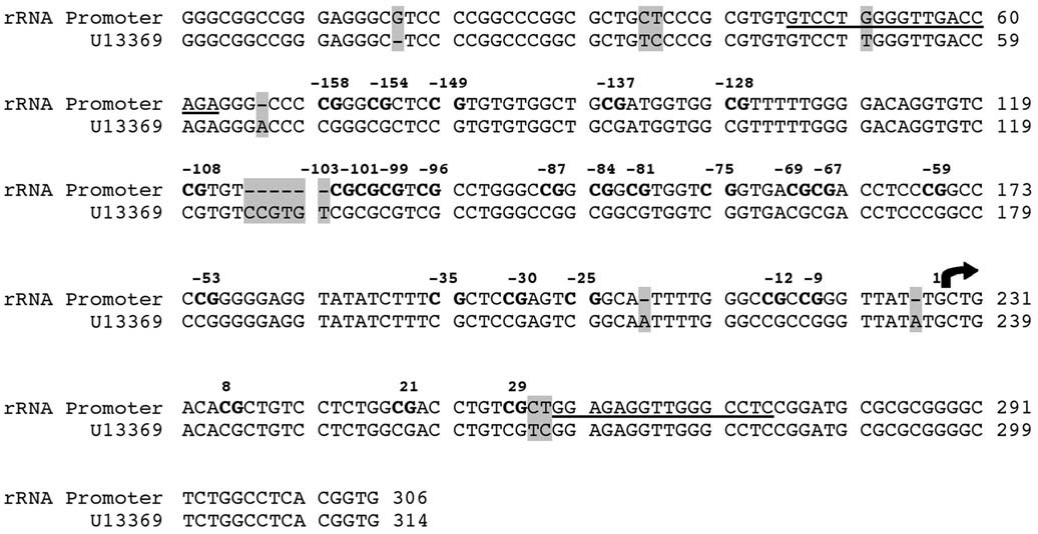
Genotyping of the rRNA promoter. The rRNA promoter sequence was identical for all suicide subjects and controls. The sequence derived from genotyping is shown above the published rRNA sequence, indicating consensus sequences for primers used for sodium bisulfite mapping (underline) and CpG dinucleotides (bold font), with locations marked relative to the transcription start site (arrow). Differences with the published rRNA sequence, U13369, are highlighted in grey, and the base pair length of each sequence is listed on the right side.

Because alterations in rRNA function may occur due to both genetic and epigenetic differences, the rRNA promoter region from each suicide subject and control was sequenced. No sequence variants were seen among subjects (**Fig. 1**; also see **Fig. S1**). When the sequence was compared to the published reference sequence for the rRNA promoter region (Genebank accession number: U13369) a few discrepancies were discovered. Notably, two CpG dinucleotides were not found in the sequenced DNA (**Fig. 1**). One CpG dinucleotide between the CpG dinucleotides in positions −108 and −103 was simply absent, and a second just upstream of the CpG dinucleotide in position 23 was found to be a C/T nucleotide substitution. Thus, of the 28 CpG dinucleotides in the published sequence, 26 CpG dinucleotides were present in the samples. As all subjects in our study were of French-Canadian origin, a population with a well established founder effect [35], it is likely that these differences reflect population-specific variants with regards to the reference sequence. For each subject, sequences overlapping the region targeted by primers after bisulfite conversion were identical to the published sequence, except for the presence of a G/T conversion in the forward primer that was present in all subjects, thus eliminating potential primer bias between subjects in sodium bisulfite mapping.

### The rRNA Promoter is Hypermethylated in the Hippocampus of Suicide Subjects

**Figure 2:**
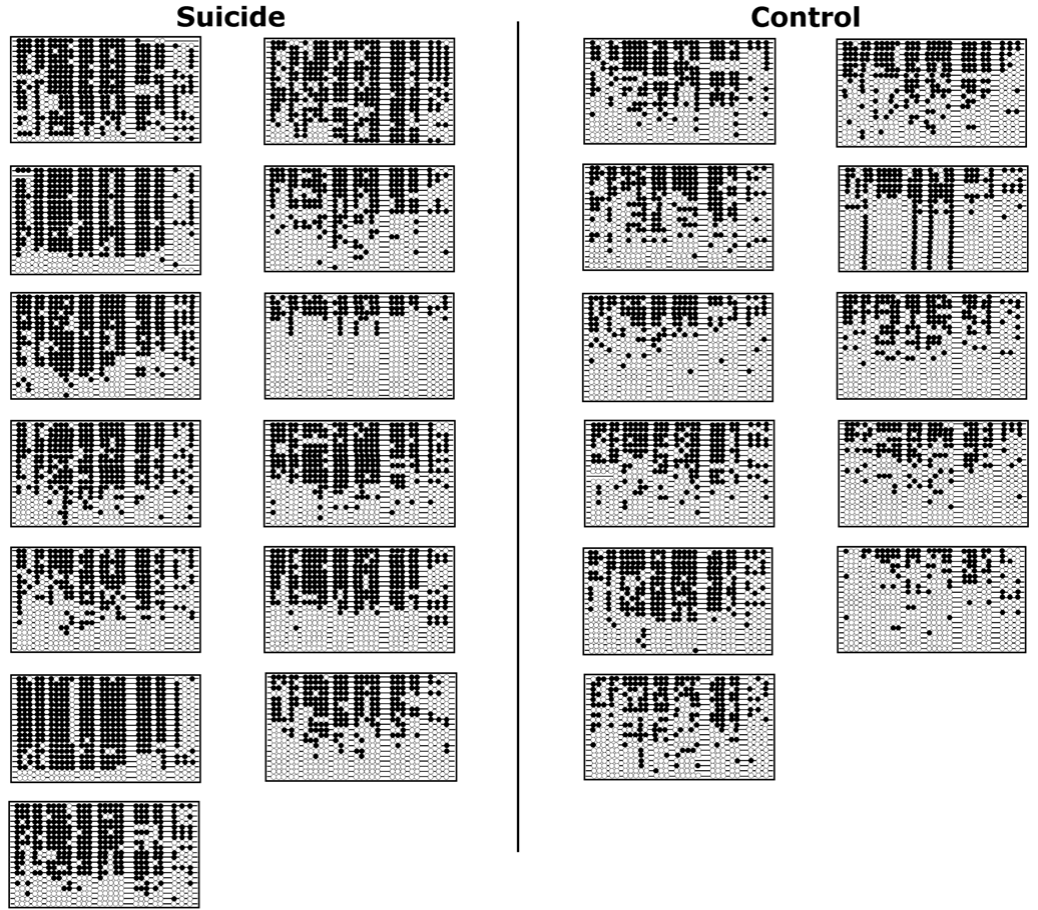
Sodium bisulfite mapping of the rRNA promoter in suicide subjects and controls. Twenty clones were sequenced for each suicide subject (left side) and control (right side), from 2 to 3 independent PCR reactions. Each line represents one clone. Circles representing CpG dinucleotides follow the 5′ to 3′ order of the rRNA promoter sequence for methylated CpG dinucleotides (filled circles), and unmethylated CpG dinucleotides (open circles).

**Figure 3:**
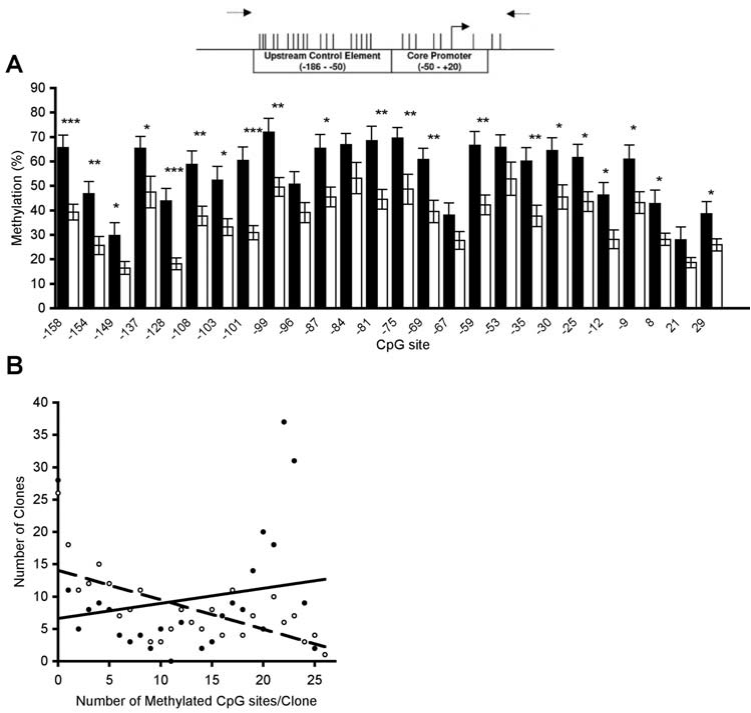
Hypermethylation of the rRNA promoter in suicide subjects relative to controls. (A) (above) Vertical lines indicate locations of CpG dinucleotides on the rRNA promoter relative to the transcription start site, indicated with the solid arrow, with primer pairs used for bisulfite mapping marked by dashed arrows. (below) Average percentage of methylation for each CpG site, for suicide subjects (N = 13; black bars) and controls (N = 11; white bars). Data are expressed as mean ± S.E.M. *, P<0.05; **, P<0.01; ***, P<0.001, measured by ANOVA. (B) Multiple regression analysis of the number of methylated CpGs per clone and the number of clones shows a significant interaction between suicide subjects (20 clones × 13 subjects, N = 260 total clones; filled circles) and controls (20 clones × 11 subjects, N = 220 total clones; open circles). There are 26 circles per group, as clones are grouped according to methylation status.

DNA methylation can affect expression via specific methylation of distinct CpG sites [29] and/or via regional site-independent changes in the overall density of methylation [30]. Previous work in cell culture on the state of human rRNA promoter methylation showed regional differences in methylation between active and inactive rRNA promoters [32]. To determine whether the pattern of methylation of single nucleotides and/or the overall methylation of rRNA differed between suicide subjects and controls, the rRNA promoter was examined at single nucleotide resolution by sodium bisulfite mapping (**Fig. 2**). The rRNA promoter was heavily methylated throughout the promoter and 5′ regulatory region in the hippocampus of suicide subjects in comparison with that of controls (F(1) = 191.04, P<0.0001, **Fig. 2** **,** **3A**). Twenty-one out of 26 CpG sites were significantly more methylated in suicide subjects compared to controls, whereas no CpG was more methylated in controls relative to suicide subjects (F(25) = 11.01, P<0.001; **Fig. 3A**). An analysis of the effect size for each CpG site revealed that these 21 sites did not differ in the magnitude of the methylation difference between groups at each CpG site (P's>0.05).

A greater number of sequenced clones were hypermethylated in the suicide subjects, whereas control subjects showed a greater number of hypomethylated clones (**Fig. 3B**). An analysis of the regression slopes between groups revealed a significant (F(1) = 7.33, P<0.01) interaction between the number of methylated CpG sites per clone for suicide subjects compared to controls. These data indicate a dramatic increase in the ratio of methylated to unmethylated clones among suicide subjects across most CpG sites.

**Figure 4:**
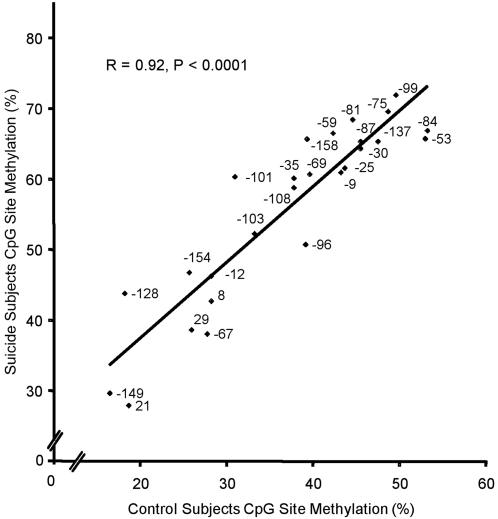
Site-independent hypermethylation of the rRNA promoter in suicide subjects. Positive correlation between suicide and control rRNA promoter methylation percentage across CpG sites (N = 26), showing a conserved hypermethylation in suicide subjects throughout the promoter region. Each data point is labeled with the position of each CpG dinucleotide in the rRNA promoter, relative to the transcription start site.

To determine whether the state of methylation of specific CpG methylation sites differed across the rRNA promoter between suicide subjects and controls or whether all CpG sites differed similarly between the two groups, the relationship between groups of the average percentage of methylation for each CpG site was investigated. There was a strong linear relationship between the means of the two groups (R = 0.92, P<0.00001; **Fig. 4**) demonstrating a similar difference in the state of methylation of all CpG sites in between the groups. No single CpG site stood out as being particularly different between the groups suggesting no site selectivity of methylation in the rRNA of suicide subjects. Instead, the overall level of methylation throughout the promoter and regulatory region differed between the groups. These data showing a lack of site-specificity are consistent with our recent analysis of the state of methylation of rRNA genes in cultured cells [32].

### Specificity of Hippocampal rRNA methylation by Analysis of Cerebellum rRNA and Genome-Wide Methylation

**Figure 5:**
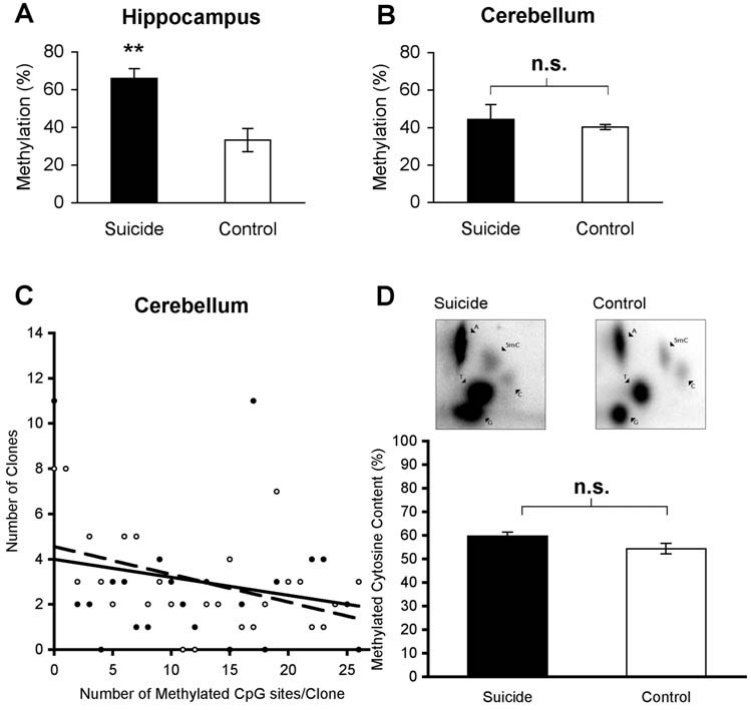
Anatomical and Genomic specificity of rRNA hypermethylation. Average percentage of rRNA promoter methylation for selected subjects with large methylation differences in the hippocampus (A) and in the cerebellum (B) of suicide subjects (N = 4, black bars) and controls (N = 4, white bars) for the same subjects. Data are expressed as mean ± S.E.M. **, P<0.01, measured by unpaired t-test. (C) Multiple regression analysis shows a similar negative relationship between the number of methylated CpGs per clone and the number of clones in cerebellum samples from suicide subjects (20 clones × 4 subjects, N = 80 total clones; filled circles) and controls (20 clones × 4 subjects, N = 80 total clones; open circles). There are 26 circles per group, as clones are grouped according to methylation status. (D) (above) Representative images of genome-wide methylation in the hippocampus for a suicide subject and a control, showing cytosine (C) and 5-methylcytosine (5 mC) content used for nearest neighbor analysis. (below) Quantification of the percentage of methylcytosine, following the formula: [(5-methylcytosine) × 100]/(5-methylcytosine + cytosine), shows no difference between suicide subjects (N = 13, black bar) and controls (N = 11, white bar) in genome-wide levels of methylation (P>0.05), measured by unpaired t-test.

To examine the anatomical specificity of the differences in rRNA methylation between suicides and controls in the hippocampus, rRNA promoter methylation was examined in the cerebellum, a region not primarily associated with psychopathology. Individuals from the suicide subjects group who showed large differences in hippocampal rRNA promoter methylation by contrast to those in the control group (t(6) = 4.12, P<0.01; **Fig. 5A**) were selected to test whether these differences would be conserved in another brain region. In contrast to the hippocampus, there was no significant difference in the percentage of methylated CpG sites between suicide subjects and controls in the cerebellum (t(6) = 0.55, P = 0.6; **Fig. 5B**). There was no significant correlation between levels of methylation in the hippocampus and those in the cerebellum (R = 0.11, P = 0.78), showing anatomical specificity in the hypermethylation of the rRNA promoter in hippocampus of suicide subjects. The interaction between the regression slopes for the number of methylated CpG sites per clone was not significant (F(1) = 0.26, P = 0.61; **Fig. 5C**), indicating that there was no difference in the ratio of unmethylated to methylated clones between groups in the cerebellum.

To determine whether the observed differences in methylation of the rRNA promoter in the hippocampus reflect genome-wide differences in methylation between suicide subjects and controls, nearest neighbor analysis of the overall percentage of methylated cytosines was performed for each subject. Nearest neighbor analysis revealed no significant difference between suicide subjects and controls in the overall percentage of methylated cytosines (t(22) = 0.54, P = 0.59), and there was no significant correlation between the percentage of rRNA promoter methylation and genome-wide methylation (R = 0.07, P = 0.78), revealing specificity in the regulation of the rRNA promoter by methylation in the suicide brain (**Fig. 5D**).

### rRNA Methylation Does Not Vary With Psychiatric Diagnoses

Next, the relationship between methylation and psychiatric diagnoses was examined (**Table 1**). Mood disorders and substance abuse disorders are risk factors for suicide and have also been linked to alterations of DNA methylation in several genes [24], [25], [36], [37]. There were no significant differences within the suicide subjects or the controls when the overall percentages of rRNA methylation of those with mood disorders were compared to those without mood disorders as well as among those with substance abuse disorders compared to those without substance abuse disorders (all P's>0.05).

### rRNA Expression is Downregulated in Suicide

**Figure 6:**
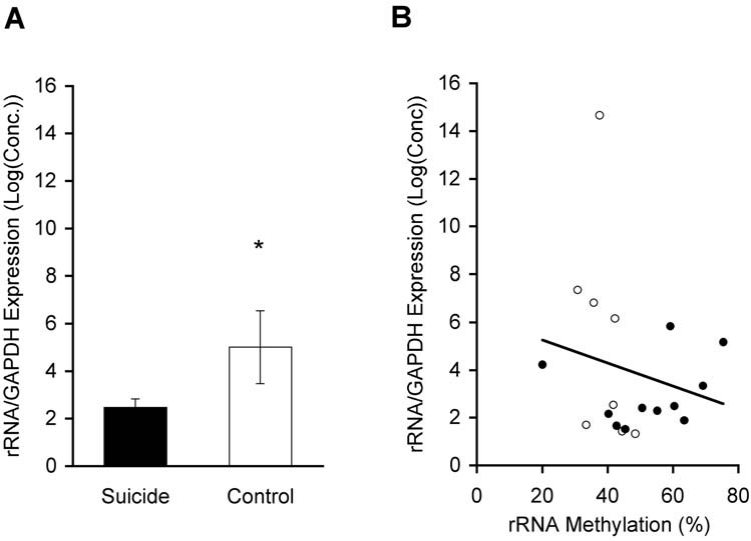
rRNA expression is downregulated in suicide. (A) Suicide subjects (N = 16, black bar) showed significantly less rRNA expression than controls (N = 9, white bar); Data expressed as mean ± S.E.M. *, P<0.05, measured by unpaired t-test. (B) Among subjects where both methylation and expression of rRNA were analyzed, the correlation between rRNA promoter methylation percentage and expression showed a trend for an inverse relationship between methylation percentage and expression of rRNA in suicide subjects (N = 11, filled circles) and controls (N = 8, open circles).

Because it has been established in cell culture that methylation of the rRNA promoter regulates the transcription of rRNA [31]–[34], rRNA expression in suicide subjects and controls was investigated. rRNA expression was significantly higher in controls than in suicide subjects (t(23) = 2.16, P<0.05; **Fig. 6A**). Correlational analysis revealed a trend for a relationship between the overall percentage of methylation and rRNA expression (R = −0.21, P>0.05; **Fig. 6B**), indicating that rRNA expression may be regulated by methylation and additional epigenetic factors. There was no relationship between and PMI, brain pH, or age and rRNA expression (P's>0.05).

## Discussion

The data reveal evidence for DNA hypermethylation of the rRNA promoter region in the hippocampus of suicide subjects with histories of childhood abuse or severe neglect relative to controls (victims of sudden, accidental death with no history of abuse or neglect). Although our findings are largely based on correlational studies indicating an association between psychopathology and methylation, these data are consistent with growing evidence suggesting that alterations in cytosine methylation mediate biological processes associated with psychopathology [38].

Since DNA methylation is a highly stable mark, the bond between a methyl group and cytosine ring being one of the most stable chemical bonds [18], the differences in methylation are unlikely to be a consequence of conditions immediately preceding death or during the postmortem interval. No reaction which could spontaneously demethylate 5-methylcytosine in DNA has ever been described. Our data indicate that post mortem pH does not affect DNA methylation (CE POM VD MJM MS and GT, unpublished observations). Indeed, post-mortem interval, brain pH, or age did not differ between suicide subjects and controls.

The increase in hippocampal rRNA promoter methylation among suicide subjects appears to occur in the absence of obvious site-specific effects on particular CpG sites. The results are consistent with data in cell culture showing that transcriptionally active rRNA promoters are completely unmethylated while transcriptionally inactive molecules are completely methylated in a promoter-wide manner without any obvious site selectivity [32]. In contrast to the situation in humans reported here and previously reported in human cells in culture, the situation in mouse is different. In the mouse, a site-specific change in methylation is sufficient to mediate silencing of the rRNA promoter [31].

Importantly, the changes in rRNA promoter methylation do not reflect a genome-wide change in methylation, as nearest neighbor analysis revealed no differences in overall levels of methylation in suicide subjects relative to controls. Moreover, this difference in the methylation of the rRNA promoter shows anatomical specificity. When the rRNA methylation status for subjects with large methylation differences in hippocampus was assessed in the cerebellum, suicide subjects and controls showed similar levels of rRNA methylation. In contrast to the hippocampus, the number of methylated CpG sites observed per clone was similar between suicide subjects and controls in the cerebellum. As a part of the brain not primarily associated with neuroplastic changes influencing psychopathology, this result indicates that rRNA methylation differences between the groups are specific to the hippocampus.

In addition to the difference in methylation, suicide subjects showed impaired hippocampal rRNA expression compared to controls. The decrease in gene expression was associated with increased methylation of the rRNA promoter sequence, as indicated by a trend for a linear correlation between the overall percentage of methylation and gene expression. Although a trend was evident, the results do not exclude other known epigenetic mechanisms influencing rRNA gene expression. For example, in cultured cells pharmacological manipulation of the acetylation status of histone 4 influences rRNA expression [31], [39].

In this study, we selected suicide subjects with a history of early childhood neglect/abuse. Childhood abuse in humans is associated with decreased hippocampal volume, as well as with cognitive impairments [9]. This influence of childhood adversity and epigenetic aberrations later in life supports the hypothesis that, similar to what was observed in rodents [19], [20], [40], early childhood events in humans alter epigenetic markings in the brain. It is tempting to speculate that epigenetic processes mediate effects of social adversity during childhood on the brain that persist into adulthood and are known to enhance suicide risk [41], [42].

Epigenetic differences might be driven by genetic differences as well as by other environmental and dietary factors. All suicide subjects in our study displayed the same genomic sequence in the rRNA promoter region examined. Additional genetic polymorphisms in other regions of the rRNA gene, including among individual rRNA gene clusters, may play a role in rRNA function. The structure and length of rRNA gene clusters varies between individuals [43], [44], however, the relationship between rRNA promoter methylation or rRNA expression and this additional level of organizational complexity in rRNA is less clear. Genetic abnormalities in rRNA gene cluster organization are associated with increased rRNA methylation during cellular senescence [45]. However, the lack of difference between groups in the cerebellum argues against such genetic differences among these individuals. Another factor that may influence the methylation status of individuals is medication prescribed in the treatment of psychiatric disorders. The mood stabilizing effect of sodium valproate, a potent histone deacetylase (HDAC) inhibitor known to indirectly influence DNA methylation [46], [47] via chromatin modification and used in the treatment of bipolar disorder, and the noted antidepressive effect of S-adenosyl methionine, a methyl donor in the DNA methylation reaction and an inhibitor of active demethylation [48] , suggest a role for DNA methylation in mood regulation [49]. Although none of the subjects in the present study had been treated with these pharmacological agents, the possibility of epigenetic regulation by other pharmacological interventions should not be discounted. Our data do not exclude these alternative hypotheses.

In summary, these data reveal increased promoter-wide methylation of the rRNA promoter as well as decreased rRNA gene expression in suicide subjects. The results of the psychological autopsy suggest a developmental origin, however, at time this is speculation based on samples that differ along a wide range of experiential and potentially genetic dimensions. To date, our data are merely consistent with the hypothesis that early life events can alter the epigenetic status of genes that mediate neural functions, and thus contribute to individual differences in the risk for suicide.

## Materials and Methods

### Subjects and Sample Preparation

Hippocampal samples obtained from the Quebec Suicide Brain Bank included: 13 suicide subjects and 11 controls matched for post-mortem interval (PMI), gender, and age (**Table 1**). Cerebellar samples were also obtained for 4 of the same suicide subjects and 4 of the same controls as those used for hippocampal analysis. An additional 6 hippocampal samples consisting of 5 suicide subjects and 1 control were also obtained from the Quebec Suicide Brain Bank for RNA expression analysis, to compensate for the removal of 5 subjects (2 suicide and 3 controls) due to low RNA quality. All samples were from male suicide and control subjects of French-Canadian origin that were processed as previously described [50]. Samples were dissected at 4°C and stored in plastic vials at −80°C until analysis. All samples were processed and analyzed blind to demographic and diagnostic variables. Possible confounders that were examined included PMI, brain pH, and age at death of the donor (**Table 1**). This study was approved by our local Institutional Review Board and signed informed consent was obtained from next of kin.

To be included in this study, all subjects had to die suddenly, with no medical or paramedic intervention. Suicide as the cause of death was determined by the Quebec Coroner's Office. Psychiatric diagnoses were obtained by means of the SCID I [51] and SCID II [52] interviews adapted for psychological autopsies, which is a validated method to reconstruct psychiatric history by means of extensive proxy-based interviews, as outlined elsewhere [41]. In addition, to be considered in this study, all suicide subjects and none of the controls had to have a positive history of childhood abuse or severe neglect, as determined by structured interviews using the Childhood Experience of Care and Abuse (CECA) [53] questionnaire adapted for psychological autopsies, as described elsewhere [54].

### Genotyping of the rRNA promoter region

Genomic DNA was extracted (DNeasy, Qiagen) according to the manufacturer's protocol. Primers for PCR were directed against the rRNA gene promoter (Genebank accession number U13369) using the following sequences: 5′-GTG TGT CCC GGT CGT AGG-3′; antisense: 5′-GTC ACC GTG AGG CCA GAG-3′. Primers were selected on the basis that they covered a 400bp region that included the region selected for sodium bisulfite analysis, including the regions covered by sodium bisulfite primers. The resulting PCR products for each subject were sequenced bidirectionally using the forward and the reverse primer on an ABI 3100 genetic analyzer (Applied Biosystems) and following the manufacturer's instructions. Genetic variation was assessed throughout the rRNA promoter region used for bisulfite analysis by alignment of genomic DNA with the previously published rRNA gene promoter sequence using freely available software (CLC Workbench, CLC bio).

### Sodium Bisulfite Mapping of DNA Methylation Status

Genomic DNA was extracted (DNeasy, Qiagen) and sodium bisulfite conversion of genomic DNA was performed as previously described [55], [56] for 13 suicide subjects and 11 controls for hippocampal samples and for 4 suicide subjects and 4 controls for cerebellum samples. Primers for PCR were directed against the rRNA gene promoter using the following sequences: sense: 5′-GTT TTT GGG TTG ATT AGA-3′; antisense: 5′-AAA ACC CAA CCT CTC C-3′ [32]. Because the primers did not contain CpG dinucleotides, methylated and unmethylated sequences amplified with equal efficiency. The resulting product was excised, purified, subcloned, and transformed (TA cloning kit, Invitrogen). Individual clones were extracted and sequenced (CEQ 8800, Beckman-Coulter) according to the manufacturer's protocol. Twenty clones were sequenced for each subject from 2 to 3 independent PCR reactions. To ensure that the bisulfite conversion was complete, only clones in which all cytosine residues in non-CpG dinucleotides had been converted to thymidine were included in the analysis.

### Nearest Neighbor Quantification of Methylated Cytosine Content

Genome-wide levels of 5-methylcytosine were quantified as previously described [57]. Briefly, genomic DNA from the same subjects as those used for bisulfite analysis was subjected to MBoI restriction enzyme digestion (recognition sequence: NGATCN), incubated with a ^32^P-labelled oligonucleotide, loaded onto TLC phosphocellulose plates, and separated by chromatography. Reactions were repeated in triplicate. The intensities of 5-methylcytosine and cytosine spot densities were analyzed using a PhosphoImager screen followed by Image Quant image analysis. For each subject, levels of methylated cytosine were tabulated as a percentage of total cytosine content, following the formula: [(5-methylcytosine) × 100]/(5-methylcytosine + cytosine).

### RNA Extraction and Quantitative Reverse Transcription PCR (qRTPCR) of rRNA Gene Expression

RNA extraction was performed using Trizol (Invitrogen) followed by Dnase I treatment, and cDNA conversion was performed using random hexamer primers (Invitrogen) according to the manufacturer's instructions (Roche Molecular Biochemicals). The subjects were the same as those used for the bisulfite mapping study, however, RNA samples from subjects with RNA Integrity Numbers (RINs) lower than 5.0 or brain pH less than 6.0 were excluded from analysis (N = 2 suicide subjects and N = 3 control subjects), consistent with previously described criteria for exclusion [50]. Because of the low number of remaining subjects and preliminary data indicating a trend for lower rRNA expression in the suicide subjects used for bisulfite analysis, we included an additional 5 suicide subjects (total N = 16) and 1 control subject (total N = 9) for rRNA expression analysis. Primers were directed against the rRNA gene using the following sequences: sense; 5′-TTC TCT AGC GAT CTG AGA GGC GT-3′, antisense; 5′-TAC CAT AAC GGA GGC AGA GAC AGA-3′ and GAPDH (Genebank accession number NM_002046) using the following sequences: sense; 5′-GAA GGT GAA GGT CGG ACT C-3′, antisense; 5′-GAA GAT GGT GAT GGG ATT TC-3′. A master mix, containing the cDNA, 10 mM Tris-Cl, 50 mM KCl, 2.0 mM MgCl2, 0.2 mM dNTPs, 5X SYBR Green I Solution, HotStart Taq DNA polymerase (Superarray Bioscience Corporation), and 0.2 µM of the sense and antisense primers, were loaded into LightCycler capillaries (Roche Molecular Biochemicals). For the rRNA gene, the qRTPCR protocol (LightCycler Software 3.5, Roche Molecular Biochemicals) consisted of initial HotStart Taq DNA polymerase activation cycle (15 min, 95°C, with a temperature transition rate of 20°C/sec) followed by 36 cycles of denaturation (30 sec, 95°C, with a temperature transition rate set at 20°C/sec), annealing (30 sec, 55°C, with a temperature transition rate set at 20°C/sec) and elongation (30 sec, 72°C, with a temperature transition rate set at 20°C/sec). A single fluorescence reading was acquired at the end of each elongation step. Subsequently, the PCR products were melted using the following program: 95°C with a temperature transition rate of 20°C/sec, 65°C, with a temperature transition rate of 20°C/sec followed by 95°C, with a temperature transition rate of 0.1°C/sec. The presence of a single melting peak followed by analysis on 1.5% agarose gel confirmed product specificity. For the GAPDH gene, the above procedure was identical except for annealing temperatures of 51°C and 61°C during the PCR and melting steps, respectively. Reactions were repeated in triplicate. Reactions were also carried out in the absence of reverse transcriptase to verify the absence of genomic DNA contamination. To determine the relative concentrations of rRNA gene expression, a standard curve of 10-fold serial dilutions of a mixture of each of the sample cDNA was used to plot the relative Ct value for each gene on the y-axis and the amount of cDNA used on the x-axis. To calculate the fold-change, the relative amount of rRNA product was divided by the relative amount of GAPDH for each subject.

### Statistical Analyses

Statistical analyses were carried out using Statview (Cary, NC). For DNA methylation analysis, a factorial ANOVA was carried out with the percentage of methylation as the dependent variable and group (suicide subjects and controls) as the between groups factor. The data were then subjected to Bonferroni Post-hoc analysis to examine methylation status between groups across all CpG sites. A standardized effect size and associated 95% confidence interval of the methylation differences between suicide subjects and controls was calculated for each CpG site using the difference between group means divided by a pooled standard deviation corrected for bias, according to previously described methods [58], [59]. The analysis of the relationship between DNA methylation at each CpG site between suicide subjects and controls was conducted using linear regression, as were analyses of the relationships between DNA methylation, expression, PMI, brain pH, and age. To identify possible diagnostic variables influencing methylation status, factorial ANOVA followed by Bonferroni Post-hoc comparisons were used to compare groups of subjects with different clinical diagnoses. For nearest neighbor as well as rRNA expression analysis, unpaired t-tests were used to examine differences between the suicide and control groups. Data from these statistical analyses are presented as mean ± SEM. Statistical significance was determined at P≤0.05.

## Supporting Information

Figure S1 The published rRNA promoter sequence U13369 is followed by the sequencing results for each subject for the region examined by sodium bisulfite mapping. The base pair length of each sequence is listed on the right side and the consensus sequence at the bottom of the sequencing results.
(0.38 MB PDF)

## References

[1] Turecki G (2001). Suicidal behavior: is there a genetic predisposition?. Bipolar Disord.

[2] Mann JJ (2002). A current perspective of suicide and attempted suicide.. Ann Intern Med.

[3] Roy A, Segal NL, Sarchapone M (1995). Attempted suicide among living co-twins of twin suicide victims.. Am J Psychiatry.

[4] Roy A, Segal NL, Centerwall BS, Robinette CD (1991). Suicide in twins.. Arch Gen Psychiatry.

[5] Seguin M, Lesage A, Turecki G, Bouchard M, Chawky N (2007). Life trajectories and burden of adversity: mapping the developmental profiles of suicide mortality.. Psychol Med.

[6] Brezo J, Paris J, Barker ED, Tremblay R, Vitaro F (2007). Natural history of suicidal behaviors in a population-based sample of young adults.. Psychol Med.

[7] Fergusson DM, Horwood LJ, Lynskey MT (1996). Childhood sexual abuse and psychiatric disorder in young adulthood: II. Psychiatric outcomes of childhood sexual abuse.. J Am Acad Child Adolesc Psychiatry.

[8] Widom CS, DuMont K, Czaja SJ (2007). A prospective investigation of major depressive disorder and comorbidity in abused and neglected children grown up.. Arch Gen Psychiatry.

[9] Vythilingam M, Heim C, Newport J, Miller AH, Anderson E (2002). Childhood trauma associated with smaller hippocampal volume in women with major depression.. Am J Psychiatry.

[10] Schloss P, Henn FA (2004). New insights into the mechanisms of antidepressant therapy.. Pharmacol Ther.

[11] Dwivedi Y, Mondal AC, Rizavi HS, Faludi G, Palkovits M (2006). Differential and brain region-specific regulation of Rap-1 and Epac in depressed suicide victims.. Arch Gen Psychiatry.

[12] Dwivedi Y, Mondal AC, Rizavi HS, Conley RR (2005). Suicide brain is associated with decreased expression of neurotrophins.. Biol Psychiatry.

[13] Odagaki Y, Garcia-Sevilla JA, Huguelet P, La Harpe R, Koyama T (2001). Cyclic AMP-mediated signaling components are upregulated in the prefrontal cortex of depressed suicide victims.. Brain Res.

[14] Sequeira A, Klempan T, Canetti L, ffrench-Mullen J, Benkelfat C (2007). Patterns of gene expression in the limbic system of suicides with and without major depression.. Mol Psychiatry.

[15] Tochigi M, Iwamoto K, Bundo M, Sasaki T, Kato N (2008). Gene expression profiling of major depression and suicide in the prefrontal cortex of postmortem brains.. Neurosci Res.

[16] Szyf M, McGowan P, Meaney MJ (2008). The social environment and the epigenome.. Environ Mol Mutagen.

[17] Razin A (1998). CpG methylation, chromatin structure and gene silencing-a three-way connection.. Embo J.

[18] Razin A, Riggs AD (1980). DNA methylation and gene function.. Science.

[19] Weaver IC, Cervoni N, Champagne FA, D'Alessio AC, Sharma S (2004). Epigenetic programming by maternal behavior.. Nat Neurosci.

[20] Champagne FA, Weaver IC, Diorio J, Dymov S, Szyf M (2006). Maternal care associated with methylation of the estrogen receptor-alpha1b promoter and estrogen receptor-alpha expression in the medial preoptic area of female offspring.. Endocrinology.

[21] Grayson DR, Jia X, Chen Y, Sharma RP, Mitchell CP (2005). Reelin promoter hypermethylation in schizophrenia.. Proc Natl Acad Sci U S A.

[22] Veldic M, Kadriu B, Maloku E, Agis-Balboa RC, Guidotti A (2007). Epigenetic mechanisms expressed in basal ganglia GABAergic neurons differentiate schizophrenia from bipolar disorder.. Schizophr Res.

[23] Dong E, Guidotti A, Grayson DR, Costa E (2007). Histone hyperacetylation induces demethylation of reelin and 67-kDa glutamic acid decarboxylase promoters.. Proc Natl Acad Sci U S A.

[24] Abdolmaleky HM, Cheng KH, Faraone SV, Wilcox M, Glatt SJ (2006). Hypomethylation of MB-COMT promoter is a major risk factor for schizophrenia and bipolar disorder.. Hum Mol Genet.

[25] Kuratomi G, Iwamoto K, Bundo M, Kusumi I, Kato N (2007). Aberrant DNA methylation associated with bipolar disorder identified from discordant monozygotic twins.. Mol Psychiatry advance online publication, 1 May 2007 (doi: 10.1038/sj.mp.4002001).

[26] Iwamoto K, Bundo M, Yamada K, Takao H, Iwayama-Shigeno Y (2005). DNA methylation status of SOX10 correlates with its downregulation and oligodendrocyte dysfunction in schizophrenia.. J Neurosci.

[27] Ding Q, Markesbery WR, Chen Q, Li F, Keller JN (2005). Ribosome dysfunction is an early event in Alzheimer's disease.. J Neurosci.

[28] Ding Q, Markesbery WR, Cecarini V, Keller JN (2006). Decreased RNA, and Increased RNA Oxidation, in Ribosomes from Early Alzheimer's Disease.. Neurochem Res.

[29] Comb M, Goodman HM (1990). CpG methylation inhibits proenkephalin gene expression and binding of the transcription factor AP-2.. Nucleic Acids Res.

[30] Nan X, Campoy FJ, Bird A (1997). MeCP2 is a transcriptional repressor with abundant binding sites in genomic chromatin.. Cell.

[31] Santoro R, Grummt I (2001). Molecular mechanisms mediating methylation-dependent silencing of ribosomal gene transcription.. Mol Cell.

[32] Brown SE, Szyf M (2007). Epigenetic programming of the ribosomal RNA promoter by MBD3.. Mol Cell Biol.

[33] Ghoshal K, Majumder S, Datta J, Motiwala T, Bai S (2004). Role of human ribosomal RNA (rRNA) promoter methylation and of methyl-CpG-binding protein MBD2 in the suppression of rRNA gene expression.. J Biol Chem.

[34] Majumder S, Ghoshal K, Datta J, Smith DS, Bai S (2006). Role of DNA methyltransferases in regulation of human ribosomal RNA gene transcription.. J Biol Chem.

[35] Charbonneau H, Guillemette A, Légaré J, Desjardins B, Landry Y (1987). Naissance d'une population—les Français établis au Canada au XVIIe siècle..

[36] Bonsch D, Lenz B, Kornhuber J, Bleich S (2005). DNA hypermethylation of the alpha synuclein promoter in patients with alcoholism.. Neuroreport.

[37] Bleich S, Lenz B, Ziegenbein M, Beutler S, Frieling H (2006). Epigenetic DNA hypermethylation of the HERP gene promoter induces down-regulation of its mRNA expression in patients with alcohol dependence.. Alcohol Clin Exp Res.

[38] Tsankova N, Renthal W, Kumar A, Nestler EJ (2007). Epigenetic regulation in psychiatric disorders.. Nat Rev Neurosci.

[39] Hirschler-Laszkiewicz I, Cavanaugh A, Hu Q, Catania J, Avantaggiati ML (2001). The role of acetylation in rDNA transcription.. Nucleic Acids Res.

[40] Meaney MJ, Szyf M (2005). Environmental programming of stress responses through DNA methylation: life at the interface between a dynamic environment and a fixed genome.. Dialogues Clin Neurosci.

[41] Dumais A, Lesage AD, Alda M, Rouleau G, Dumont M (2005). Risk factors for suicide completion in major depression: a case-control study of impulsive and aggressive behaviors in men.. Am J Psychiatry.

[42] Turecki G (2005). Dissecting the suicide phenotype: the role of impulsive-aggressive behaviours.. J Psychiatry Neurosci Nov.

[43] Stults DM, Killen MW, Pierce HH, Pierce AJ (2008). Genomic architecture and inheritance of human ribosomal RNA gene clusters.. Genome Res.

[44] Caburet S, Conti C, Schurra C, Lebofsky R, Edelstein SJ (2005). Human ribosomal RNA gene arrays display a broad range of palindromic structures.. Genome Res.

[45] Machwe A, Orren DK, Bohr VA (2000). Accelerated methylation of ribosomal RNA genes during the cellular senescence of Werner syndrome fibroblasts.. Faseb J.

[46] Detich N, Bovenzi V, Szyf M (2003). Valproate induces replication-independent active DNA demethylation.. J Biol Chem.

[47] Milutinovic S, D'Alessio AC, Detich N, Szyf M (2007). Valproate induces widespread epigenetic reprogramming which involves demethylation of specific genes.. Carcinogenesis.

[48] Detich N, Hamm S, Just G, Knox JD, Szyf M (2003). The methyl donor S-Adenosylmethionine inhibits active demethylation of DNA: a candidate novel mechanism for the pharmacological effects of S-Adenosylmethionine.. J Biol Chem.

[49] McGowan PO, Kato T (2008). Epigenetics in mood disorders.. Environ Health Prev Med.

[50] Sequeira A, Turecki G (2006). Genome wide gene expression studies in mood disorders.. OMICS.

[51] Spitzer RL, Williams JB, Gibbon M, First MB (1992). The Structured Clinical Interview for DSM-III-R (SCID). I: history, rationale, and description.. Arch Gen Psychiatry.

[52] First MB, Spitzer RL, Gibbon M, Williams JBW (1995). The structured clinical interview for DSM-III-R personality disorders (SCID-II). Part I: Description.. J Personal Disord.

[53] Bifulco A, Brown GW, Harris TO (1994). Childhood Experience of Care and Abuse (CECA): a retrospective interview measure.. J Child Psychol Psychiatry.

[54] Zouk H, Tousignant M, Seguin M, Lesage A, Turecki G (2006). Characterization of impulsivity in suicide completers: clinical, behavioral and psychosocial dimensions.. J Affect Disord.

[55] Clark SJ, Harrison J, Paul CL, Frommer M (1994). High sensitivity mapping of methylated cytosines.. Nucleic Acids Res.

[56] Frommer M, McDonald LE, Millar DS, Collis CM, Watt F (1992). A genomic sequencing protocol that yields a positive display of 5-methylcytosine residues in individual DNA strands.. Proc Natl Acad Sci U S A.

[57] Ramsahoye BH (2002). Nearest-neighbor analysis.. Methods Mol Biol.

[58] Hedges LV, Olkin I (1985). Statistical methods for meta-analysis..

[59] Kirk RE (2007). Effect magnitude: A different focus.. J Statistical Planning and Inference.

