# Supplementary figures and images for: Promoter-Wide Hypermethylation of the Ribosomal RNA Gene Promoter in the Suicide Brain
Source: PLoS One. 2008 May 7;3(5):e2085. doi: 10.1371/journal.pone.0002085 (PMC2330072; doi:10.1371/journal.pone.0002085)

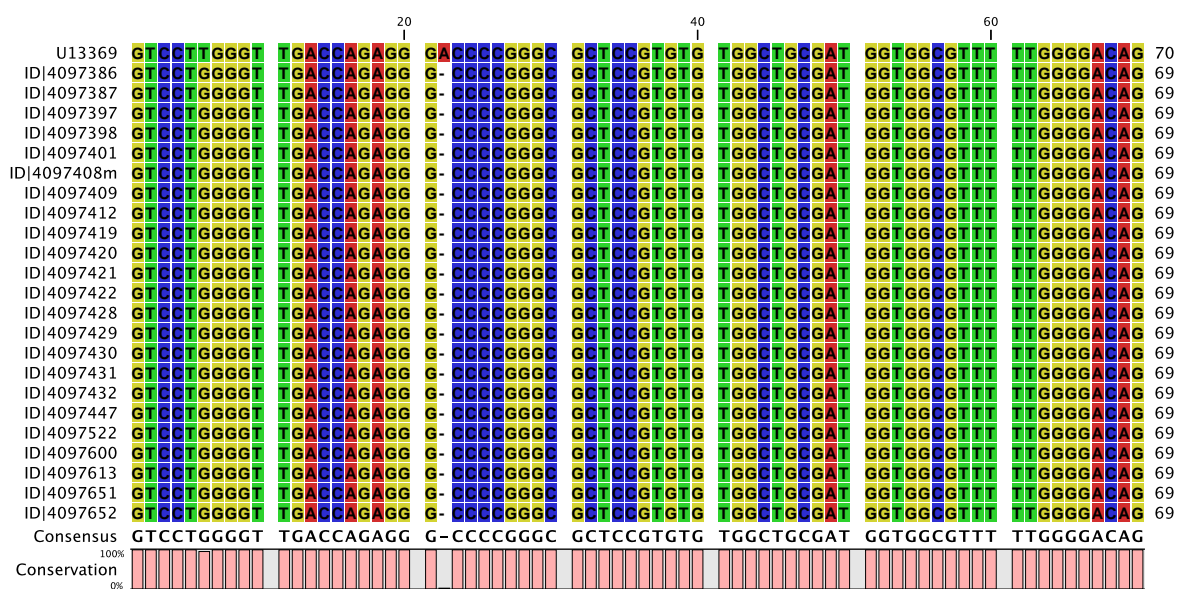

Supplement: Figure S1 — The published rRNA promoter sequence U13369 is followed by the sequencing results for each subject for the region examined by sodium bisulfite mapping. The base pair length of each sequence is listed on the right side and the consensus sequence at the bottom of the sequencing results. (0.38 MB PDF) [file pone.0002085.s001.pdf]
